# Causal diagramming for assessing human system risk in spaceflight

**DOI:** 10.1038/s41526-024-00375-7

**Published:** 2024-03-19

**Authors:** Erik Antonsen, Robert J. Reynolds, Jacqueline Charvat, Erin Connell, Avalon Monti, Devan Petersen, Nicholas Nartey, Wilma Anton, Ahmed Abukmail, Kristina Marotta, Mary Van Baalen, Daniel M. Buckland

**Affiliations:** 1 Center for Space Medicine, Department of Emergency Medicine, Baylor College of Medicine, Houston, TX USA; 2 KBR, Houston, TX USA; 3 Leidos Innovations, Houston, TX USA; 4 JES Tech, Houston, TX USA; 5 University of Houston-Clear Lake, Houston, TX USA; 6 NASA Johnson Space Center, Houston, TX USA; 7 Duke University, Durham, NC USA

**Keywords:** Risk factors, Epidemiology, Research management, Aerospace engineering, Biomedical engineering

## Abstract

For over a decade, the National Aeronautics and Space Administration (NASA) has tracked and configuration-managed approximately 30 risks that affect astronaut health and performance before, during and after spaceflight. The Human System Risk Board (HSRB) at NASA Johnson Space Center is responsible for setting the official risk posture for each of the human system risks and determining—based on evaluation of the available evidence—when that risk posture changes. The ultimate purpose of tracking and researching these risks is to find ways to reduce spaceflight-induced risk to astronauts. The adverse effects of spaceflight begin at launch and continue throughout the duration of the mission, and in some cases, across the lifetime of the astronaut. Historically, research has been conducted in individual risk “silos” to characterize risk, however, astronauts are exposed to all risks simultaneously. In January of 2020, the HSRB at NASA began assessing the potential value of causal diagramming as a tool to facilitate understanding of the complex causes and effects that contribute to spaceflight-induced human system risk. Causal diagrams in the form of directed acyclic graphs (DAGs) are used to provide HSRB stakeholders with a shared mental model of the causal flow of risk. While primarily improving communication among those stakeholders, DAGs also allow a composite risk network to be created that can be tracked and configuration managed. This paper outlines the HSRB’s pilot process for this effort, the lessons learned, and future goals for data-driven risk management approaches.

## Introduction

Human spaceflight is a high-risk endeavor. The Challenger and Columbia disasters acquainted engineers, mission planners, and the public with the risk stemming from high-energy phases of flight, however, these same groups often have limited insight into other spaceflight-induced risks to humans^1^. The National Aeronautics and Space Administration (NASA) currently tracks approximately 30 human health and performance risks that crewmembers face during spaceflight, all of which derive from the 5 hazards of human spaceflight^2–4^. These human system risks are evaluated against 8 design reference missions (DRMs), which are mission templates for assessing risk posture relevant to current missions and to expected future missions^3–5^. DRMs include short-duration missions in low Earth orbit, missions to the lunar orbit or the lunar surface, and excursions to Mars that can last up to 3 years. Although efforts have been made to understand and mitigate these risks, significant uncertainty remains regarding how much risk crewmembers will face as mission durations increase and flights extend further from Earth^6^.

In addition to protecting astronauts during any given mission, NASA is responsible for the clinical care of astronauts after their missions, and, to some extent, after their careers have ended. Research into spaceflight-induced human system risks has traditionally been organized into specialized risk “silos,” which was advantageous for characterizing risk when NASA’s Human System Risk Board (HSRB) risk portfolio management was being established and risk records were being developed. However, because spaceflight-induced risk to the human system is multifactorial and cumulative, this siloed perspective challenges communication among experts from different disciplines who struggle to contextualize the contribution of risk from outside their specialty area. Differing mental models of the sources of risk, the factors contributing to risk, and the relationships between those factors impede clear and informed recommendations regarding the urgency and prioritization of addressing risk within the limited NASA resources. Cross-disciplinary research efforts have emerged to address the issue of ‘siloing’, however, better methods of characterizing and representing the complex interactions between risks are still needed^7^. The lack of systematic approaches to characterize the complexity and multifactorial contributions to human system risk also exacerbates the challenge of conveying that spaceflight-induced deterioration of human capability must be adequately addressed when designing and building spacecraft systems. The inherent complexity of human system risks and spacecraft systems necessitates new approaches to understanding and communicating risk so that investments in risk reduction in one area do not create unintended consequences (i.e., elevated risk in other mission areas)^2,8^.

Establishing a common understanding of the state of these risks and avoiding unintended consequences is an ongoing challenge for at least 2 reasons. First, the field of human spaceflight is still nascent, and the evidence base that describes how humans respond to the spaceflight environment is still being assembled. The strength of conclusions that can be drawn from available evidence are often not sufficient to reliably bound risk for mission planners and program managers. Second, spaceflight-induced effects on human health and performance result from a complex interplay of factors that range from the genetic, molecular, and cellular responses of an individual, to the social interactions between multiple individuals who make up a team, to the ability of that team to engage successfully with vehicle systems to resolve anomalies. Assessing human system risk involves many areas of expertise, imparting a level of complexity that makes it difficult to form a complete picture of risk. Previous attempts to capture these interactions through graphical representations have been inadequate^9,10^. The challenge of addressing systemic complexity is daunting and novel approaches for mapping, communicating, and analyzing that complexity are lacking in the human spaceflight community. The HSRB risk stakeholders must prioritize decisions based on a limited (and potentially nonintegrated) set of information about the relative importance of the various risks and the key factors that contribute to them. Risk stakeholders also lack a way to align the different mental models of the key risk factors and relationships that are intuitively held by different subject matter experts (SMEs). This leads to communication failures within organizations, and between the HSRB and the operational programs that must consider human system risk when designing vehicles and planning missions. Because no common terminology exists for the risk framework and assessments, this complicates the process of reaching agreement between stakeholders regarding the relative importance of the many risk factors, the value of proposed monitoring or research, and the prioritization of system components needed to reduce spaceflight-induced risk to humans. The HSRB posited that a map of interactions that is supported by available evidence and input from SMEs could help stakeholders interpret the larger complex picture of risk, and this would lead to improved mitigation of the risks.

Graphs have been used extensively in other areas of health and medicine to link related concepts^11–15^. For example, toxicology studies often graph adverse outcome pathways to conceptualize physiologic issues and their complex inter-relationships^16^. Once the conceptual framework of the graphs are arranged, standardized representation and terminology are developed to enable a systematic, repeatable, and quantitative process for analyzing the complex interactions of factors of importance to the scientific and clinical communities^17–19^. NASA’s HSRB piloted an approach based on similar principles using causal diagrams in the form of directed acyclic graphs (DAGs) to map each of the human system risks. Initially this effort was designed to improve communication because the causal flow of each risk, when communicated in a visual format such as a DAG, facilitates a common understanding among stakeholders. This graphical format allows clinicians and researchers to identify the “part” of the risk they are attempting to characterize or mitigate and where additional efforts could be allocated. This enables a more fluid discussion of how those risk characterization and mitigation activities can impact mission-level outcomes of concern to other stakeholders, such as program managers. The goal of this pilot project was to evaluate if causal diagrams can improve the process of managing human system risks at NASA. The lessons learned from this project indicate that a larger network of the individual risk diagrams could be created to support greater insight into the complex interactions between risks. More importantly, the DAGs also form the basis for a graph schema that can facilitate systematic and quantitative data-driven approaches for identifying the gaps in knowledge and spaceflight capability that are critical for reducing risk during future exploration missions. This paper recounts the work done to date and explores the advantages and limitations of this approach.

## Methods

### Directed acyclic graphs

**Fig. 1:**
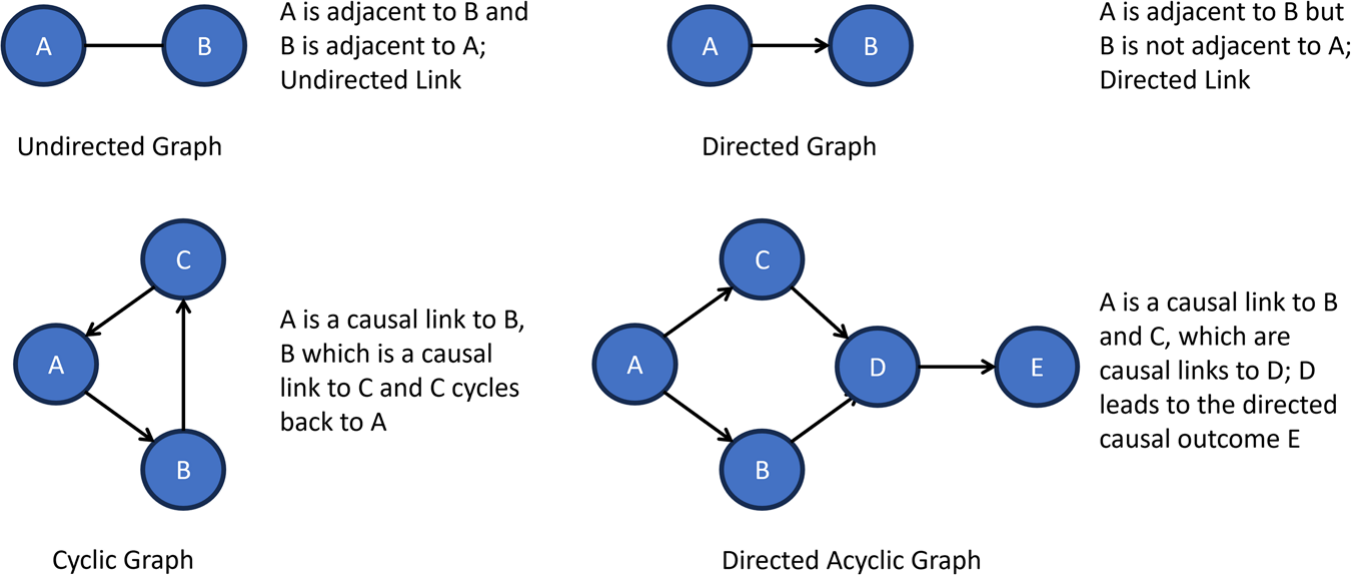
Examples of graph data structures.

A graph data structure is composed of a set of *vertices* (nodes), and a set of *edges* (links). Each edge represents a link between 2 nodes, indicating the nodes are *adjacent*. A graph can only have one type of link, either directed or undirected, making it either a directed graph or an undirected graph (see Fig. 1 for examples). For example, if the link between 2 nodes *a* and *b* is undirected (no arrow), then *a* and *b* are said to be adjacent (*a* is adjacent to *b* and *b* is adjacent to *a*). However, if the link between *a* and *b* is directed by an arrow pointing from *a* to *b*, then *a* is adjacent to *b* but *b* is not adjacent to *a* (unless another directed link exits going from *b* back to *a)*. A directed graph can contain a cycle of one or more links that extend from a specific node and return to that same node. A directed graph that has no cycles is known as a directed acyclic graph (DAG)^20^.

Therefore, a DAG can be a type of network diagram that, through specific conventions in its construction, represents causality in a visual format^14,21^. Specifically, each directed arrow connecting one node to another node indicates causality. Causality here means the probability distribution of the effect variable is conditional on the value of its causal variable(s). In simpler terms, we can imagine a suspected causal dichotomous factor: i.e., the probability of an outcome is different when the factor is present than when it is absent. Table 1 shows an example of causal and non-causal relationships between 2 binary variables, A and B.

**Table 1:** Illustration of potential causal relationships between factor A and outcome B

	Probability outcome P (B = 1 | A) when…	
Value of A	A not a cause of B	A is a cause of B
Absent	0.6	0.6
Present	0.6	0.8

As the first column in Table 1 shows, when A is not a cause of B the probability that outcome B occurs is 0.6, whether factor A is present or not. However, if A is a cause of B, then the probability of event B occurring is greater in the presence of factor A (0.8) than it is in absence of factor A (0.6). An example pertaining to spaceflight is that exposure to altered gravity (i.e., gravity less than that on Earth [1 g]) causes unloading of bones in the body, which then causes bone remodeling. This in turn can lead to skeletal fragility and an increased likelihood of bone fractures. In practice this means that the probability of observing a given amount of bone remodeling is contingent on the gravity field to which astronauts are subjected. Future space missions may include exposure to lunar (1/6 g) or Mars (3/8 g) gravity in addition to microgravity exposure in orbit and during deep space transit. This example spans from a hazard of spaceflight (altered gravity) to cellular level changes (bone remodeling) to functional changes (skeletal fragility) to an outcome (bone fracture).

### DAG derivation for human system risks

Creating DAGs for each of the human system risks requires SMEs to logically map out what factors contribute to the flow of each risk. The flow of the risk from common starting points to common ending points can be presented at an appropriate level of detail for non-experts to understand and can also be expanded in detail to be useful to the SMEs. The process, therefore, suggests 2 types of DAGs. “Narrative” DAGs convey high-level, aggregate concepts linking the key components of the causal flow to downstream effects, and these graphs are used to facilitate communication and shared mental models at a board or stakeholder meeting. Each narrative DAG has a corresponding written narrative, which is presented as a bulleted walkthrough of the DAG’s causal story. “Detailed” DAGs support the high-level “story” of the narrative DAGs and include additional detail that may help explain causal flow and gaps in knowledge. The detailed DAGs must conform to that same data schema (the graphical model that defines relationships between graph entities^22^) as the high-level data schema in the narrative DAGs. To ensure compatibility with other risk DAGs, this means that both the narrative and the detailed DAGs must adhere to strict standards for representing relationships and prescriptive terminology.

As mentioned previously, no feedback loops are permitted in DAGs. This requirement ensures that causes precede effects, and that the diagrams show a simplified flow of risk from the hazard exposures to the mission-level outcomes, enabling understanding at a high level. The requirement also ensures the creators of the DAGs articulate the most important steps that are likely to lead to the undesirable outcomes, while avoiding the excess detail that often derails effective communication about risk. If the graph creators detect a feedback loop, they must choose the most likely predecessor node and represent it earlier in the causal chain.

Spaceflight-induced human system risks have historically been conceptualized as deriving from 5 hazards that are encountered at the time of launch into space^2–4,9^. Therefore, these hazards are the starting point for the HSRB’s causal diagramming. Whether an astronaut is launched to suborbital space for a short time or to Mars for a year-long mission, exposure to these hazards induces the physiologic changes they experience during spaceflight. Physiologic changes alone may not lead to risk, instead the interaction between human crew capability—which itself may degrade during spaceflight—and the vehicle and mission systems that the crew must operate also contribute to risk.

The mission-level outcomes that represent the best return on investment for characterizing or mitigating human system risks are those that matter to 3 groups of risk stakeholders:(1) Astronauts who must accept the consequences of spaceflight, both during a mission and long after it the mission is complete; (2) NASA Program managers who decide what to include in the vehicles and systems that support human crews in space; and (3) the NASA Office of the Chief Health and Medical Officer and the Health and Medical Technical Authority who are responsible for NASA’s human system risks^4,5^. These groups must consider serious outcomes that could result from tradeoffs in resources when spaceflight vehicle resources must be severely limited^2^. For this reason, the HSRB defines mission-level outcomes (that can occur either during or after a mission) as those that rise to clinical or operational significance, including less severe outcomes such as loss of mission objectives and worst-case scenarios such as loss of crew life and loss of a mission.

### Basic requirements for human system risk DAGs

**Fig. 2:**
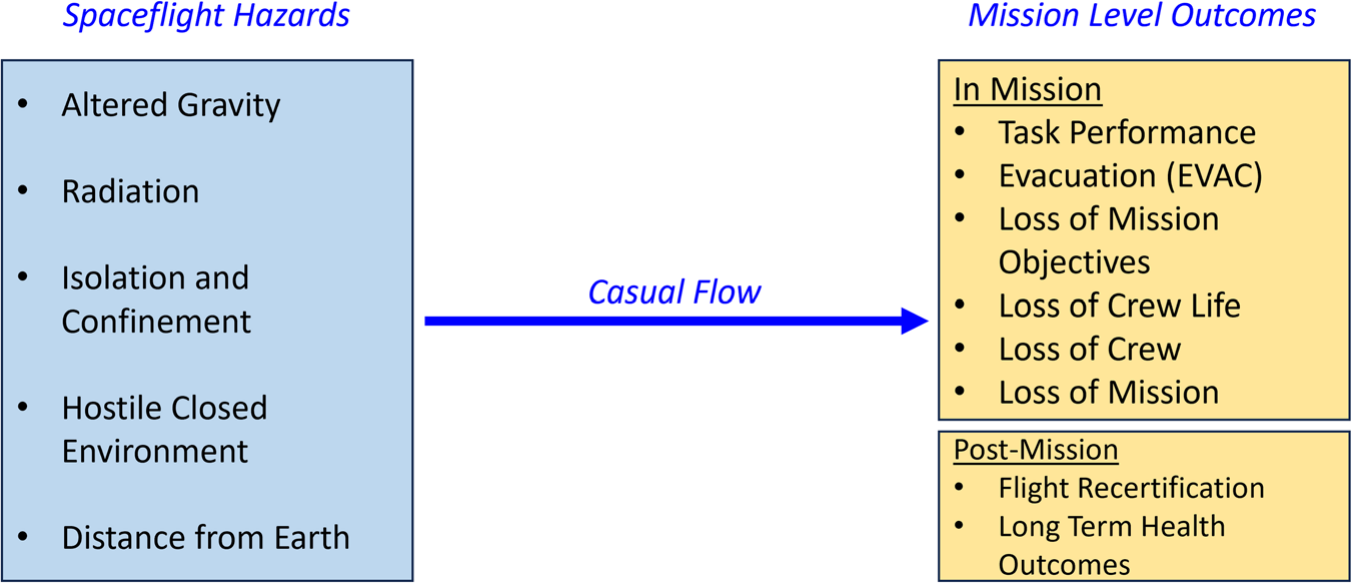
Common starting and ending points for visualizing risk is the first step to a community-wide agreement on causal flow that is supported by the available evidence^4^.

All contributing factors included in the human system risk DAGs are expected to significantly affect risk posture, as determined by either available evidence or SME concern. The structure of each DAG starts with at least one hazard and ends with at least one of the predefined mission-level outcomes (Fig. 2). In between are the nodes and edges of possible contributing factors relevant to the specific risk.

A node or connection between nodes can be included in a particular DAG if several conditions are met. First, a logical basis must exist that the node affects the risk. The HSRB has adopted a subset of 6 criteria noted in the causal guidelines outlined by Sir A. Bradford Hill to assess level of evidence and these are described in detail elsewhere^4,23,24^. The evidence on hand, as evaluated by the risk custodian team, must meet the principles of *temporality* and *analogy* as the minimum requirement to include a node in a DAG and meet the basis for a “speculative” level of evidence which is the lowest level^4^. *Temporality* states that the effect must occur after the cause (including any expected delays between them), whereas *analogy* requires known evidence of similarities between the postulated association and any other associations. Second, for a narrative DAG the magnitude of a proposed effect must be sufficient to create a measurable downstream effect on one or more adjacent nodes. These 2 requirements help limit the size and scope of the DAG to key nodes and relationships of interest. Imposing this structure also helps to ensure that key factors believed to be important to a given risk can be visually related to the outcomes of interest for risk concerns.

The basic requirements for a DAG are not sufficient to enable like-to-like comparison across risks or to reliably illustrate how risks interact: for this standardized representation of human system risks across DAGs is needed.

### Standardized DAG representation

Between the hazards and the outcomes lie the nodes and links of the causal diagram that are intended to illustrate key relationships in risk propagation. Naming and depicting nodes and the links between them in a standardized fashion ensures information across DAGs is similar, and that it is structured to align with HSRB risk configuration-managed items such as important contributing factors and countermeasures.

#### Basic drawing guidance

**Fig. 3:**
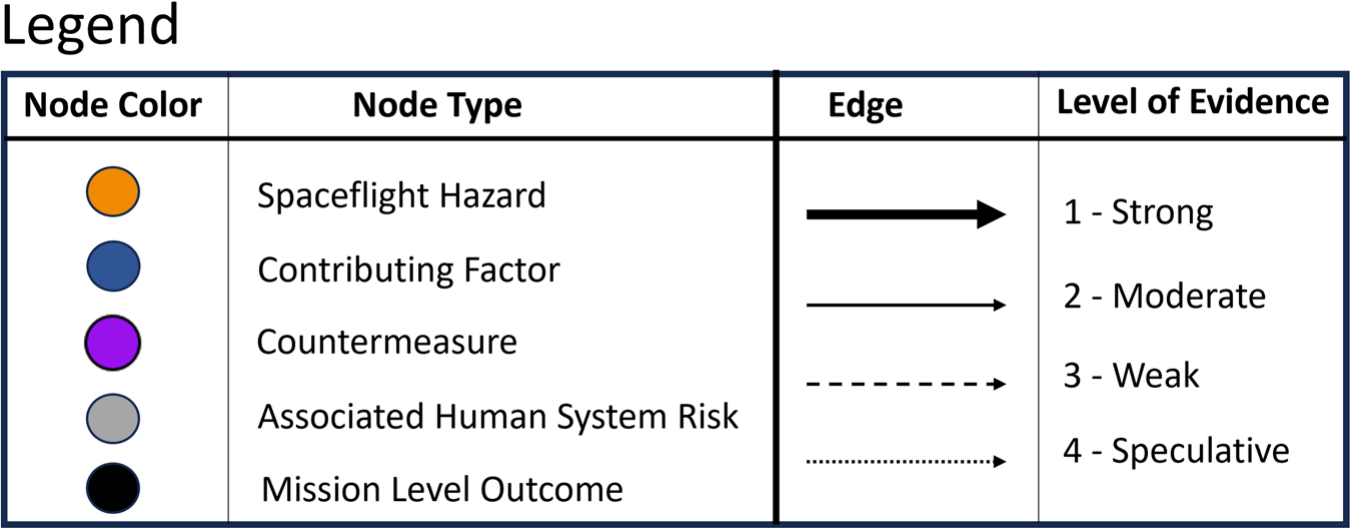
Legend for the node and edge visualizations in the DAGVision software.

Nodes in the DAGs are represented by circles, and edges are represented by arrows that are drawn from causes to effects. Exogenous nodes have one or more arrows pointing out of them and no arrows pointing into them, and they do not trace back to the hazards in the risk causal flow, whereas endogenous nodes have one or more arrows traceable to the hazards. The first DAGs were drawn using Dagitty software^25^, which is freely available on the internet, and later, DAGvision software was implemented, which is still in development at NASA. The colors and notations shown in Fig. 3 are simply a function of the DAGvision software and may be represented differently by other software.

Contributing factors is a heuristic term that describes any node that is not a hazard or a mission-level outcome. Countermeasures are a type of contributing factor that is introduced to a system specifically to mitigate risk. Spaceflight countermeasures are typically specific solutions introduced to mitigate risk, and can be technology-based (exercise devices, decision support software, radiation shielding, etc.) or non-technology-based (medications etc.). The HSRB assigns countermeasures to 3 categories: monitoring, prevention, or intervention^4^.

Prevention countermeasures prevent a deleterious impact on health or performance. For example, exercise prevents muscle atrophy and bone loss in an altered gravity environment^26–28^, and can also support behavioral health by improving mood^7^.

Intervention countermeasures are implemented in response to a recognized detriment in human health or performance that has already occurred. In the case of medical care this is often called “treatment,” whereas in the case of failure of vehicle systems this is referred to as “repair.” Both are interventions to mitigate risk resulting from a change in the human system state. Pharmaceuticals can play a preventive role or a treatment role depending on whether they are deployed before or in response to a particular medical event.

**Fig. 4:**
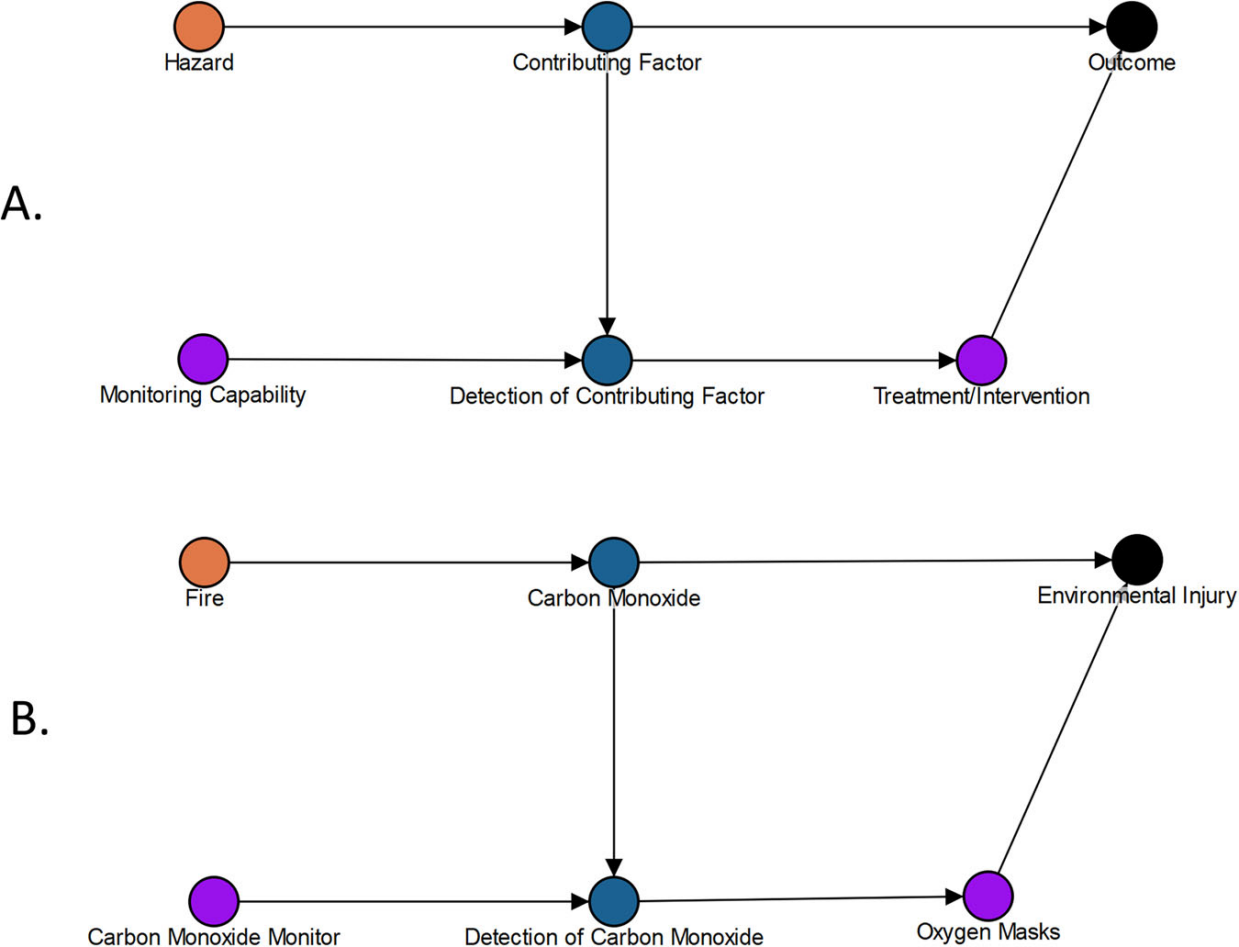
Approaches to visualizing carbon monoxide exposure as a risk. (**A**) Shows the general approach to graphing monitoring capability and (**B**) shows the spaceflight specific example for carbon monoxide exposure.

Monitoring is included as a countermeasure because many preventive or intervention actions are implemented based on monitoring of the state of the human system. If monitoring sensors and data management are not effectively designed into the space vehicle systems using human system integration processes^8,29^, the human effectively becomes the monitor themselves, i.e. injury from invisible sources like radiation, atmospheric toxins, etc. is detected only when symptoms develop, which many be too late to prevent adverse outcomes in some DRMs. These risk situations must be evident to mission planners so that they can make risk-informed decisions about what to include in vehicle systems. The inclusion of monitoring hardware and software in the vehicle depends on decisions made early in the systems engineering life-cycle^2^, so monitoring capabilities are included in DAGs as a necessary predecessor to various intervention countermeasures. Panel A of Fig. 4 shows the standard representation of the relationships between any monitoring capability, the variable being monitored, and the treatment or intervention influenced by the monitoring information collected. (Note specific node names are bolded in the discussion below to differentiate them from concepts that are not specifically referencing the nodes).

Panel B of Fig. 4 shows an example where the monitor in question is a **carbon monoxide monitor** and the variable being measured is the **carbon monoxide level** in the atmosphere of the spacecraft. The sensor that measures and the variable being measured must be present for detection to occur. Once detection occurs it influences the probability that the intervention will occur, in this case the use of **oxygen masks**. The **carbon monoxide level** directly affects the probability of **environmental injury** occurring due to the exposure: hence the causal relationship shown. However, if **oxygen masks** are used, that will also affect the probability of **environmental injury**. Therefore, both the level being detected, and the intervention are immediately adjacent to the environmental injury node, whereas the detector and detection are upstream of the intervention node. This example provides insight into the standardized representation of relationships used in all the risk DAGs.

#### Nesting of nodes

**Fig. 5:**
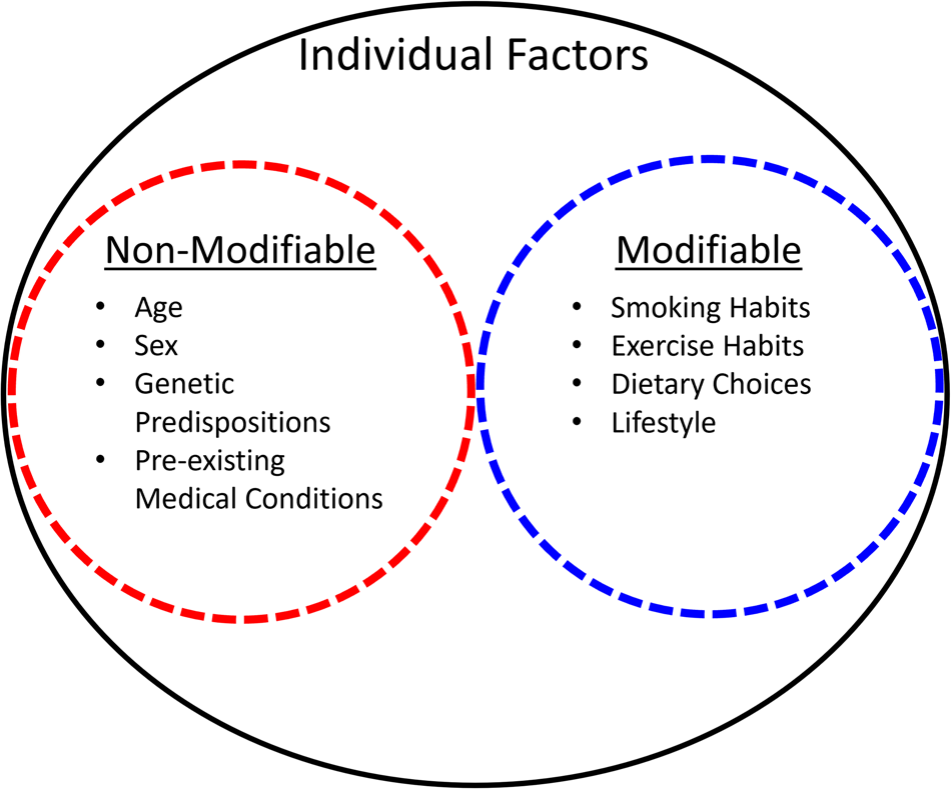
The category node “individual factors” houses 2 direct sub-nodes—modifiable factors and non-modifiable factors—that in turn house several sub-nodes shown in list format.

Standardized representation also includes the relationship between high-level nodal information and more detailed nodal information. Narrative DAGs are specifically designed to convey high-level information, and this can oversimplify some of the details beneath high-level nodes. Nodal nesting is the relationship between a high-level node and 2 or more sub-nodes. “Category” nodes are the higher-level nodes, and “sub-nodes” indicate relationships with any “category nodes.” Fig. 5 illustrates nodal nesting for a category node titled **individual factors** that has 2 sub-nodes—**modifiable factors** and **non-modifiable factors—**that have another layer of sub-nodes shown in list format.

**Fig. 6:**
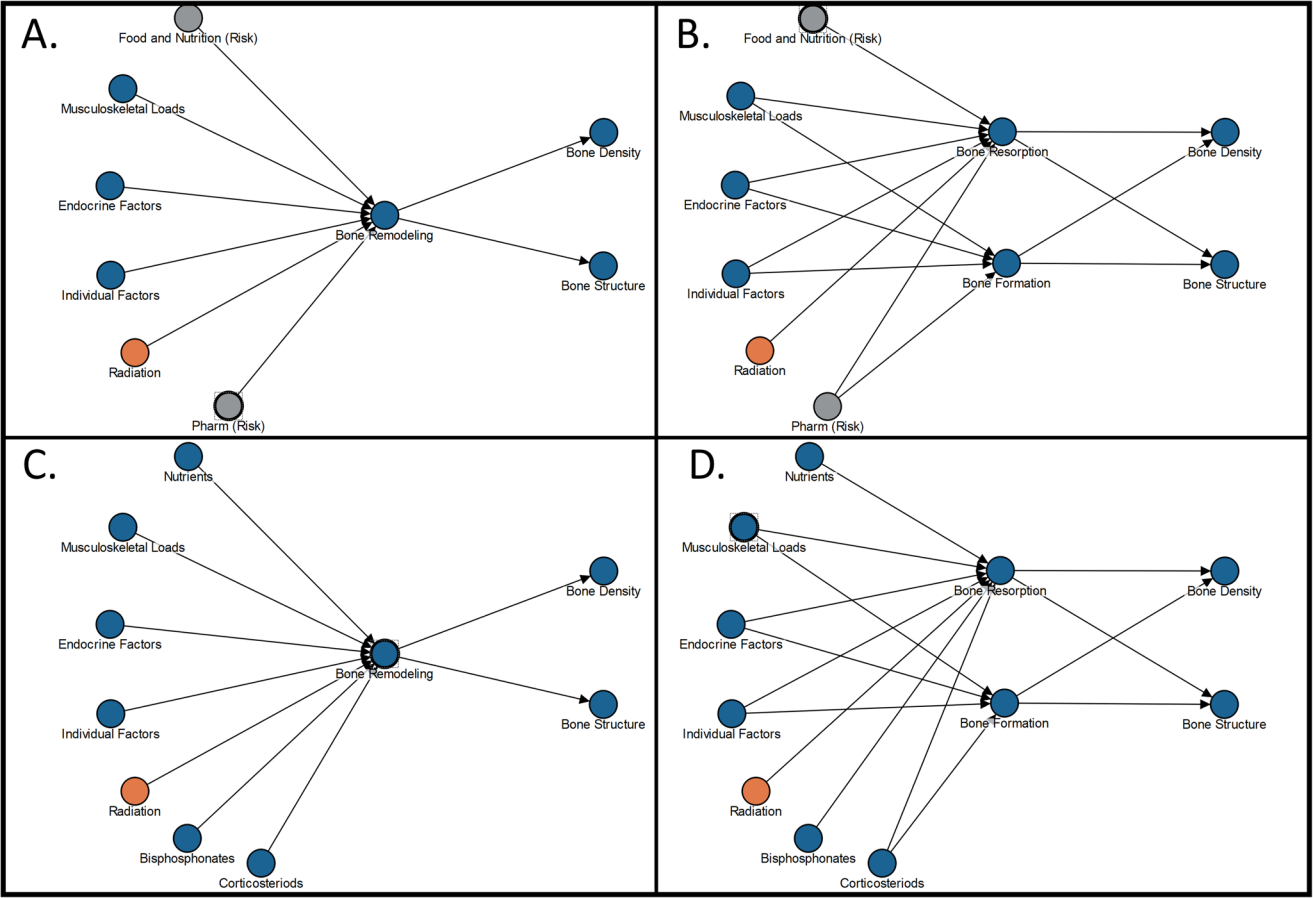
Ego network of the bone remodeling node from the bone fracture directed acyclic graphs (DAG). Panel (**A**) shows the high-level narrative visual. Panel (**B**) shows the detailed level visual where bone remodeling is subdivided onto 2 sub-nodes named bone resorption and bone formation. Panel (**C**) shows the edge-worked narrative DAG that is visually simplified using only bone remodeling. Panel (**D**) shows the edge-worked detailed DAG that is more visually complex. Pharm = Pharmacy.

The term “nesting” is used because the sub-nodes appear to be nested within the higher-level nodes. Including a category node in a DAG can help to simplify the visualization, especially in narrative DAGs. In detailed DAGs, the category node can be replaced with the sub-nodes that are relevant to the specific risk. Each arrow that goes to and from a category node must connect to at least one nested sub-node, as illustrated in Fig. 6.

Figure 6 shows an ego network for a portion of the spaceflight-induced risk of bone fracture to help illustrate this nesting concept. An ego network is a sub-graph that visualizes only a specific node of interest and any nodes that are within a specified network distance, or “radius”, of this node. In this example only the adjacent causes and adjacent effects on the **bone remodeling** node within the larger bone fracture risk DAG are visualized. Panel A shows the high-level causal hypothesis including the category node **bone remodeling**. A more detailed breakdown of **bone remodeling** into the nested sub-nodes **bone formation** and **bone resorption** is shown in the detailed level DAG (panel B). The 2 gray nodes in panel A and panel B represent an influence on bone remodeling from **food and nutrition (risk)** and **pharmacy (risk)**. However, the more detailed panel B shows that **food and nutrition (risk)** only cause changes in **bone resorption** and not **bone formation**, whereas the **pharmacy (risk)** causes changes in both **bone resorption** and **bone formation**. Panels C and D of Fig. 6 show the same visuals at a more detailed level: here salient nested nodes under the gray risk nodes are identified and replace the gray node, allowing the nested node or nodes to belong to both risks simultaneously and to demonstrate causal paths by which the risks interface. The HSRB used the term “edge work” to describe the process of visually aligning the nodes that overlap risks and ensuring that those connections do not inadvertently create cycles. For example, the blue node titled **nutrients in** Fig. 6C, D replaces the gray **food and nutrition (risk)** node in Fig. 6A, B because **nutrients** is a key node in the food and nutrition risk DAG that is known to affect **bone remodeling** through effects on osteoclastic activity^30^. In the case of the pharmacy risk, specific medications such as **bisphosphonates** are known to affect osteoclastic activity thereby affecting **bone resorption**^31,32^. Prolonged use of **corticosteroids** can affect both osteoblast and osteoclast activity and therefore affects both **bone formation** and **bone resorption**^33^ All 4 of these visuals are equivalent, but each serves a different communication purpose, and illustrate the differences between narrative and detailed visuals as well as edge-worked visuals.

#### Harmonized and neutral terminology

Individual risk DAGs can be combined into a larger DAG where nodes appear in multiple DAGs and connect the separate networks into a single network. However, for software to represent this effectively, these nodes must be named using a common lexicon. Before DAGs were implemented different groups of SMEs used at least 6 terms, including microgravity, gravitational force, weightlessness, partial gravity, to describe the **altered gravity** hazard. The use of different terminology introduces confusion in discussions among the human health and performance community at NASA and would make combining risk networks impossible if not resolved.

Harmonization is an intentional process intended to ensure that different groups of experts use the same name and definition for the same concepts. The HSRB reviewed and approved a set of harmonized terminology for use in the DAGs and for risk discussion more broadly. The risk custodian teams, who maintain the DAGs, can propose new terminology if the concept they seek to include is not already represented within the harmonized list.

The language used for naming nodes should identify a random variable (i.e., neutral terminology) rather than a realized value of a random variable. For example, a node should be named **nutritional status** (a random variable) rather than **inadequate nutrition** (a realized value of the random variable **nutritional status**). This ensures maximum utility of the node in the DAG because it reflects the idea that nutritional status may exert influence on various nodes when it is either adequate or inadequate. This makes nodes both risk-independent and DRM-independent, and the risk steps therefore apply from the moment of launch to the moment of crew recovery on Earth for any mission.

#### Configuration management

An inevitable question arises from any attempt to map out the factors that are important to a human system risk: who decides what is important? This is not a trivial question because the community who evaluates and provides input to the DAGs come from a variety of disparate fields that include medicine, life sciences, pharmacology, food sciences, behavioral health, exercise physiology, engineering, human factors, and many more. The initial narrative DAG must reflect the needs of the stakeholders involved in assessing and mitigating risk across all these domains. The risk custodian team assigned to each risk, which includes SMEs from operations, research, and epidemiology at NASA, and representatives from the HSRB and other SMEs as needed confer to create the basic DAG. The HSRB provides a forum where 100–200 members of the NASA human health and performance community review and provide input to each of the risk assessments, and since January 2020, this has included a review and discussion of the DAG for each risk. Each risk update goes through the HSRB configuration-management process that includes a formal, tracked comment period and a comment resolution process^4^. Any disagreements are discussed and resolved either by the board or by the board chair, based on evidence standards used by the HSRB^4,24^. In this way, the narrative DAGs and their write-ups receive crowd-sourced feedback that ensures their causal stories adhere to the most current evidence-based knowledge available regarding human health and performance in spaceflight.

In considering new evidence that may challenge the structure of the DAG, approaches are being developed to evaluate whether or not the current DAG structure is consistent with new data. A proposed set of procedures for DAG validation was investigated using a subset of the bone fracture risk DAG and four datasets of animal studies that analyzed bone remodeling in altered gravity environments^34^. Tests of expected marginal correlation and conditional independencies derived from the DAG indicate that the rodent data largely supported the structure of the diagram. Principles for evaluating incongruencies between the independencies and the DAG structure were articulated to help interpret the reasons for any mismatch^34^. These approaches help guide risk custodians analysis for validation of DAGs in a structured and repeatable manner.

The HSRB only configuration manages the narrative DAG and accompanying written narrative for each risk. DAGs that depict a more detailed view of each risk are not subject to the configuration management process because of the excessive amount of detail and time this would take at the HSRB.

### Applications of DAGs

#### Evolving DAGs

**Fig. 7:**
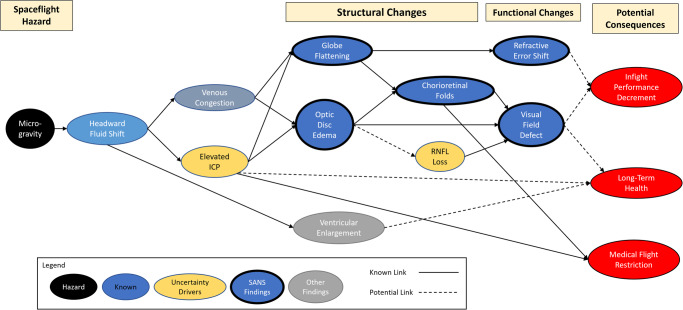
The initial directed acyclic graphs for the risk of spaceflight associated neuro-ocular syndrome (SANS) that was accepted by the Human Systems Risk Board in April 2020. Metadata was assigned to the nodes through color and border differentiation and a legend illustrates the different features of specific nodes. Additionally, solid lines represent a strong level of evidence and dotted lines represent a weak level of evidence (i.e., the strength of the evidence that supports the specific assertion of causal connection).

Once the causal flow in a DAG is agreed upon, metadata can be assigned to the nodes or the edges to allow visualization of relevant parameters. For example, the level of the evidence that is available to support any specific claim of causality within a DAG can be illustrated by varying the appearance of the connecting edges between the relevant nodes. Figure 7 shows the initial DAG for the spaceflight associated neuro-ocular syndrome (SANS) risk that was approved at the HSRB on April 23, 2020. This DAG was created prior to instituting standardized representation, harmonized and neutral terminology, and interface mapping with other human system risks. Various visual aspects of the nodes and edges are differentiated to enhance understanding of the risk story. (This image was created in Microsoft PowerPoint rather than in DAGvision).

In this visualization, the term “microgravity” was used for the hazard that is now named “altered gravity.” Dotted lines represent areas where the evidence base is weak and solid lines represent connections with a strong evidence base to support the hypothesized causal link. Node color and border thickness are used to differentiate different types of node properties. Metadata associated with the nodes and edges of any risk DAG can improve the communication and analysis of information within the network.

**Fig. 8:**
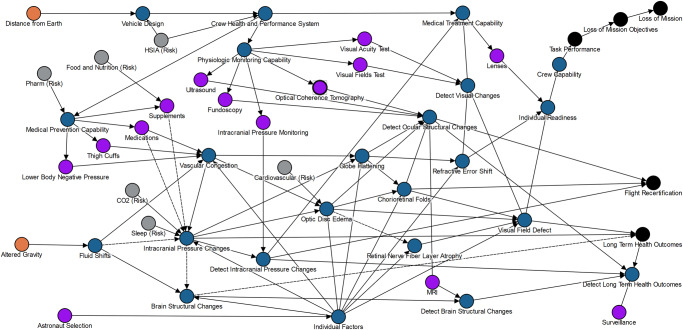
The most recent spaceflight associated neuro-ocular syndrome (SANS) risk directed acyclic graph (DAG) approved by the Human Systems Risk Board in May 2022. The DAGs are expected to change over time as additional evidence is gathered and communication needs change^39^. HSIA Human System Integration Architecture, CO_2_ Carbon Dioxide, Pharm Pharmacy, MRI Magnetic Resonance Imaging. Legend is Fig. 3.

In Fig. 8, the core story from the earlier DAG is retained but is enhanced by additional information that the SANS Risk Custodian Team incorporated from discussions within the HSRB community and refinement due to the standardization processes for DAG development. One specific change is that a pathway from the **vehicle design** and **crew health and performance system** enables inclusion of various countermeasures and the likely location of effect on the flow of risk. Because the mass, volume, power, and other allocations for the vehicles and systems are determined by **distance from Earth**, that pathway originates with that hazard and is shown at the top of the image. Trade space decisions in the design process will determine if the potential countermeasures for mitigation of a risk are included in any given mission. If not, the risk posture will likely be elevated.

Additionally, **astronaut selection** affects **individual factors** that include biologic variability of response to the spaceflight environment, and this also affects risk. This pathway is shown at the bottom of the DAG where multiple arrows are drawn to the physiologic and functional changes that occur during spaceflight. This does not imply that astronauts are intentionally selected because they have optimal responses, but rather that the chosen crewmembers will have some variation in their physiologic and functional responses.

The DAGs are expected to evolve and change as we learn more about spaceflight-induced human system risks. By establishing guidelines, implementing a configuration management process, and approving a starting version, the HSRB seeks to ensure that the causal diagrams used by NASA are the best possible reflection of the current consensus of the risk posture.

#### Connecting DAGs

**Fig. 9:**
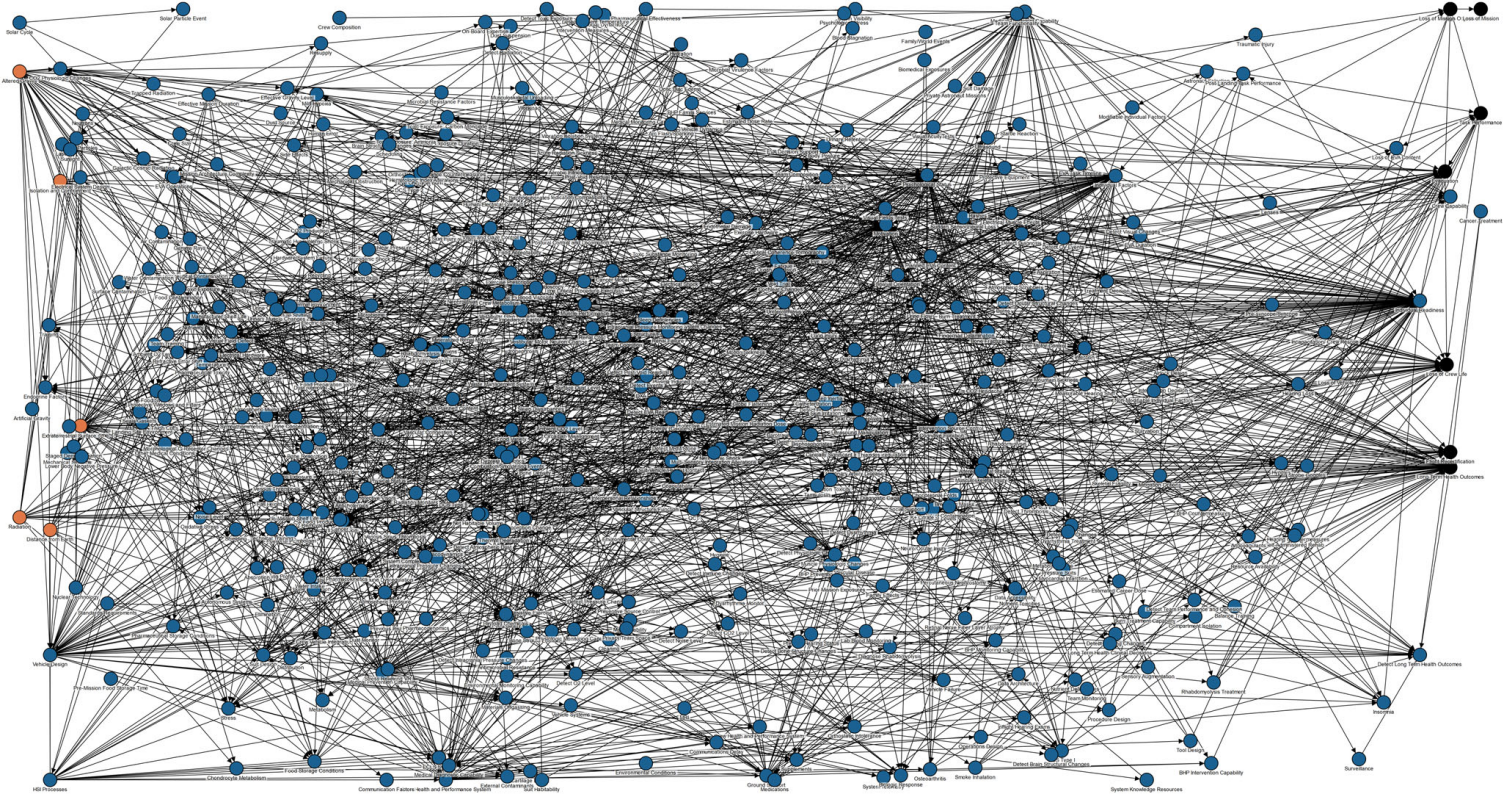
The first draft risk network created from detailed versions of the 30 individual directed acyclic graphs. Orange nodes are hazards (left edge of diagram), black nodes are mission level outcomes (right side of diagram), and blue nodes are contributing factors.

Configuration managed DAGs not only provide a communal understanding of how spaceflight-induced risks have evolved, they also help inform how the risks interact with one another. Intuitively, it seems obvious that the 30 spaceflight-induced risks are interdependent, and NASA has pursued methods to elucidate and document the interrelationships among risks and any potential cumulative effects they might pose on mission-level outcomes^9,10^. Because concept representation and terminology are standardized, and the edge work systematically replaces the gray risk placeholder nodes with blue contributing factor nodes, the set of individual DAGs can be combined through common nodes to create a risk network. Once created, this network offers the possibility of structural and computational analysis to gain insights that are unavailable in the silo approach to risk^35^. Figure 9 shows the first attempt to link the DAGs for all 30 risks into a single risk network.

This preliminary risk network image is not an official product of the HSRB but illustrates the complexity of the interrelationships and that the interpretation and analysis are beyond manual approaches. This highlights the need to develop and use computer-based network approaches to analyze the structure for insight into the effects of interactions across risks. To provide a future pathway to systematic and quantitative analysis of risk using these DAGs the eventual goal has been to use an integrated risk DAG like the one shown in Fig. 9 as a Bayesian network. In this context uncertainty must be considered in both the evaluation of new evidence and the analysis of an increasingly complex network. While this is considered future work, approaches for assessing epistemic and aleatory uncertainty in Bayesian networks are available from a variety of fields such as environmental risk assessment^36^, socio-ecological network modeling^37^, and biogeochemical modeling^38^.

## Outlook and summary

The acceptance of DAGs at the HSRB is a starting point to implementing future changes that have sufficient supporting evidence to inform additions or subtractions of nodes, or changes in the connections between nodes. The dynamic nature of DAGs also accommodates discussion and inclusion of speculative areas of concern as a potential falsifiable hypothesis in the larger network of risk. The terms dynamic and falsifiable are intentional here. All the connections within these DAGs are potentially falsifiable–they are assertions of causal interactions that are based on current understanding of the evidence. However, the human spaceflight evidence base is thin, with less than 700 humans having experienced the challenges of the spaceflight environment. As more people fly and new evidence is gathered, we expect that some connections will be disproven or modified by emerging evidence. These DAGs provide a starting place from which to document the ongoing changes in our knowledge.

The HSRB process for updating DAGs aligns with NASA’s continuous risk management processes and enables the NASA community to track how the evolving evidence from research, occupational surveillance, clinical care, and other sources elucidates each of the human system risks. Including the level of evidence scores in the updated DAGs will help program managers to make decisions^24^. For instance, distinguishing the areas of weak evidence can identify places in the DAG where new nodes should be considered and can identify gaps in knowledge or capability for addressing spaceflight-induced risk to the human system. Identifying the relationships between proposed new nodes that have pathways to mission-level outcomes can help identify the areas of research that are likely to have more valuable on investment for reducing risk and provides research scientists with a way to convey their risk concerns to program managers by pointing to a node or a connection between nodes where their research will help address a deficit in knowledge or capability. Similarly, visually identifying strong evidence supporting causal pathways can help justify design decisions for program managers who may be unsure of the relative importance of competing priorities and can help to justify the inclusion (or exclusion) of countermeasures such as certain medications, medical technologies such as ultrasound or defibrillators, or monitoring systems under consideration for spaceflight missions. In the context of operations, a strong level of evidence can help establish updates to flight rules or clinical practice guidelines that are based on knowledge that accrued since design decisions were made years before a mission.

Although this tool and approach has strengths, it also has challenges and limitations. The human health and performance community at NASA are not the only group characterizing spaceflight-induced human system risk. Other international space agencies, academic researchers, and the emerging commercial space enterprise hold sources of spaceflight data that should be considered. It is our hope that in publishing this approach alongside the recently published narrative DAGs^39^ will provide these entities with an understanding of NASA’s perspective on the human system risks and empower them to bring forward their insights to help refine and interpret risk. NASA has created an HSRB website (https://www.nasa.gov/hhp/hsrb) where all DAG related material is available, and comments can be submitted for consideration^40^.

It should also be noted that once individual DAGs are created and documented, they could impart anchoring bias on the scientific community, causing the community to rely too heavily on the first DAG developed. As such, it is important to continually challenge the story that each DAG presents when new evidence emerges, and to add and subtract from the story when sufficient evidence is gathered to justify these updates. Naming conventions for detailed levels of risk are unlikely to satisfy all stakeholders, so there must be a means of settling debate on how to name and portray nodes, categories, and relationships between nodes. This should be guided by the strength of evidence brought forward for consideration. In cases of dissent on terminology, NASA’s HSRB has the authority to determine what the “official” DAG will be until the next update to the risk occurs as part of the continuous risk management process^4^. Node naming and parameterization changes can and should be documented as risks are updated.

The appropriate level of detail for nodes can also be a source of disagreement. Some might argue that specific medical conditions should be explicitly shown in individual risk DAGs. Although this is desirable for a detailed DAG that is intended to be analyzed through computer-based data analytics, it is overwhelming for the HSRB members or program managers who must understand the high-level sources of risk. The appropriate level of detail for narrative DAGs depends on the purpose for which it is being employed and must be determined by the application. The strength of a graphical schema like this is that attributes of nodes and edges can be assigned easily and the model itself can be updated in a native graph database without losing assignments that are already in place. From a management perspective this has several advantages over relational databases. Once a schema has been created, a variety of powerful analytic algorithms can be used to gain insight from the graph itself and from data or evidence assigned to components of the graph^41^.

A common criticism of the acyclic requirement is that it ignores feedback loops that exist in the real world and are important to scientific understanding of the causal components of risk. The response to this criticism is 2-fold. First, the path through a DAG represents causal factors that influence effects *over time*. If a path leads back to its source, this will violate the coherence of the time sequence inherent in the DAG. This is true for even the simplest feedback loop: 2 nodes with directed arrows pointing at each other. For this feedback representation to be accurate, each node would be the cause of each other at precisely the same moment. Instead, we recognize that feedback loops are never simultaneous; they occur in a sequence, even if the timespan of that sequence is quite small. Second, if a cycle is truly needed to understand the science, this can be represented on the detailed DAG using time-indexed variables to visually represent the concepts as nodes that occur repeatedly over time. Using the current guidance, duration of exposure is represented as a single node in places where time is expected to contribute a clinically or operationally significant impact to the risk posture under evaluation. This approach is likely to be re-evaluated in future work.

Although the HSRB has created DAGs for each of the 30 configuration-managed risks, it is the board’s intention to continue curating the existing DAGs and developing new DAGs for any future risks yet to be defined. The standardized approach to representation and lexicon in DAGs is intended to facilitate the creation of an integrated risk network that can be used for data-driven decisions regarding risk characterization and mitigation as the evidence base for human spaceflight evolves. Developing the DAGs, DAG narratives, data model, and processes that surround the DAG creation and maintenance is the first step towards data-driven decisions. Software packages that maintain the DAGs are being developed. Ongoing work in this area includes developing algorithms for those software packages that may enable a quantitative, systematic, and repeatable approach for managing spaceflight-induced human system risk.
